# Synthesis and Characterization of Eco-Engineered Hollow Fe_2_O_3_/Carbon Nanocomposite Spheres: Evaluating Structural, Optical, Antibacterial, and Lead Adsorption Properties

**DOI:** 10.3390/nano15241850

**Published:** 2025-12-10

**Authors:** Islam Gomaa, Nikita Yushin, Mekki Bayachou, Vojislav Stanić, Inga Zinicovscaia

**Affiliations:** 1 Nanotechnology Research Centre (NTRC), The British University in Egypt (BUE), Suez Desert Road, El-Sherouk City 11837, Cairo, Egypt; islam.gomaa@bue.edu.eg; 2 Department of Nuclear Physics, Joint Institute for Nuclear Research, Joliot-Curie Str., 6, 141980 Dubna, Russia; ynik_62@mail.ru; 3 Chemistry Department, College of Arts and Sciences, Cleveland State University, Cleveland, OH 44115, USA; m.bayachou@csuohio.edu; 4 Department of Inflammation & Immunity, Lerner Research Institute, Cleveland Clinic, Cleveland, OH 44195, USA; 5 Vinča Institute of Nuclear Sciences, University of Belgrade, 11000 Belgrade, Serbia; 6 Department of Nuclear Physics, Horia Hulubei National Institute for R&D in Physics and Nuclear Engineering, 30 Reactorului Str. MG-6, 077125 Magurele, Romania

**Keywords:** carbon-doped Fe_2_O_3_, lead removal, microspheres

## Abstract

This work presents a facile mechano-thermal route for the synthesis of carbon-decorated, hollow, mesoporous α-Fe_2_O_3_ microspheres. Comprehensive characterization (XRD, XPS, FT-IR, SEM/EDX, TGA, zeta-potential) confirmed the formation of phase-pure hematite with nanoscale crystallites (~19 nm), substantial residual surface carbon (~40 wt%) consistent with Fe–O–C linkages, and a positive surface charge (+15.9 mV). The hierarchical hollow/mesoporous architecture enables fast ion transport and provides extensive interior binding sites, resulting in rapid Pb(II) uptake that reaches 92% removal in ≈15 min at pH 5.0. The adsorption follows a Langmuir isotherm (q_max_ ≈ 70.6 mg/g) and pseudo-second-order kinetics, indicative of chemisorption coupled to efficient mass transfer into internal sites. The composite also exhibits antibacterial activity against *Escherichia coli* and *Staphylococcus aureus*, demonstrating its potential for simultaneous mitigation of heavy metal contaminants and pathogens.

## 1. Introduction

Lead (Pb) ranks among the most toxic heavy metal. Despite having no known biological function and being toxic even at low concentrations, its malleability, corrosion resistance, and low melting point have led to extensive human use for thousands of years [1,2,3]. The capacity for bioaccumulation, and potential to bio-magnify through food chains make it a serious global concern for both public health and the environment [4]. Exposure affects nearly all bodily systems, with well-documented health impacts [5].

Over recent decades, significant progress has been made in understanding the sources and fate of lead in aqueous environments, alongside advances in removal technologies [6,7]. Among these, adsorption is considered a particularly effective and economical remediation method. Its advantages include operational simplicity, the availability of diverse and often low-cost adsorbents, design flexibility, high removal efficiency under various conditions, and the potential for adsorbent regeneration. Developing readily available, inexpensive, and renewable adsorbents is crucial for promoting sustainable water treatment practices [8,9,10].

Iron oxide (Fe_2_O_3_) doped carbon evacuated microspheres, derived from one of the most abundant elements in the Earth’s crust, possess favorable morphological, physicochemical, and structural properties that make them highly promising for heavy metal removal and dispersion control [11].

Hollow, mesoporous spheres combine charged or functionalized outer surfaces with internal adsorption and rapid pore-mediated ion transport. This structure results in superior heavy metal and organic pollutant uptake, alongside enhanced mass-transfer efficiency, when compared to dense nanoparticles [12]. Microspheres—typically spherical particles ranging from sub-micron to tens of micrometers (≈0.1–100 μm) in size—can be conveniently recovered from liquid media through simple operations like centrifugation, filtration, or magnetic separation. Their surfaces can be readily engineered with biomolecules or functional dyes (e.g., proteins, nucleic acids, fluorophores), establishing them as key building blocks for point-of-care diagnostic platforms. Numerous studies have reported elaborate routes to synthesize hematite microspheres; however, many protocols rely on complex processing and are not optimized for low-cost, scalable synthesis with high yield [13]. For instance, Li Xu et al. [13] reported a solvothermal synthesis of α-Fe_2_O_3_ hollow microspheres using a Fe-containing ionic liquid, 1-octyl-3-methylimidazolium tetrachloridoferrate(III) ([C_8_mim][FeCl_4_]). This ionic liquid simultaneously served as an iron source and a structure-directing agent, yielding hollow structures with enhanced electrical conductivity, photocurrent response, and photocatalytic performance. Shaowen Cao and Yingjie Zhu [14] later introduced an NaCl-assisted microwave–solvothermal route to monodisperse, mesoporous α-Fe_2_O_3_ microspheres. Their method used Fe(NO_3_)_3_ as a precursor and polyvinylpyrrolidone as a surfactant in a water/ethanol mixture. By tuning the reaction parameters, they controlled particle size and obtained microspheres with high efficiency for the photocatalytic degradation of salicylic acid. Further templated and self-assembly strategies have been proposed by Yueheng Zhang and co-workers [15], underscoring the strong interest in architecturally designed α-Fe_2_O_3_ microspheres for photocatalytic applications.

This study presents a facile mechano-thermal synthesis of carbon-doped Fe_2_O_3_ hollow spheres, which are meticulously engineered to exhibit a well-defined cubic nanocrystalline structure within a hollow spherical morphology. This unique porous architecture holds significant promise for advanced applications in adsorption, catalysis, targeted delivery systems, and the development of high-performance stable capturers. Since the formation mechanism for such shapes is not fully understood [15] their practical application has also been constrained by complex synthesis routes [13,14,15] and costly reagents. The optimized strategy presented in this work effectively overcomes these limitations. The properties of produced carbon-doped Fe_2_O_3_ hollow spheres were characterized using a set of instrumental techniques, and their adsorption capacity for lead was evaluated under different experimental conditions, including pH, contact time, and initial lead concentration).

## 2. Experimental

### 2.1. Chemicals and Reagents

Ferric chloride anhydrous (FeCl_3_, ≥98%, Sigma-Aldrich (Darmstadt, Germany)) and citric acid anhydrous (C_6_H_8_O_7_, ≥99.5%, Fisher Scientific (Fisher Scientific, UK)) were used as received. Deionized water (Milli-Q grade) served as the solvent throughout the experiment. No further purification or treatment was applied to the chemicals.

### 2.2. Synthesis of the Molecular Precursor and α-Fe_2_O_3_/C Hollow Spheres

The molecular precursor for Fe_2_O_3_ nanoparticle synthesis was prepared via a green mechano- thermal procedure [16]. Iron (III) chloride and citric acid in a 1:3 molar ratio were ground in a mortar with a pestle until a homogeneous fine powder was obtained. During grinding, 10 mL of Milli-Q water was added dropwise until the mixture formed a paste and a faint acetic-acid odour was observed [17]. The paste was transferred to 100 mL Milli-Q water and stirred at 400 rpm. The suspension was neutralized by the dropwise addition of 1.0 M NaOH and continuously stirred for 2 h. The resulting mixture was separated by centrifugation (9000 rpm, 10 min). The obtained precipitate was redispersed in 100 mL Milli-Q water, transferred to a Teflon-lined autoclave, and hydrothermally treated (100 °C, 24 h) to nucleate microsphere frameworks. The product was then vacuum-dried (100 °C, 12 h) to yield a red precursor powder (Fe-p) for further characterization. Elemental analysis for the proposed composition [Fe(C_6_H_7_O_7_)·3H_2_O] was as follows: Found: C, 24.3; H, 3.9; Fe, 19.53%. Calculated: C, 25.20; H, 3.88; Fe, 20.4%. Finally, the precursor (Fe-p) was calcined in static air (400 °C, 5 °C/min ramp, 2 h) to crystallize the α-Fe_2_O_3_/C microspheres.

### 2.3. Adsorbent Characterization

Elemental analysis (CHN) was done using Vario El-Elementar Analyzer. Powder X-ray diffraction (XRD) for phase identification and crystallographic analysis of the precursor and Fe_2_O_3_/C was performed on a Malvern Panalytical Empyrean 3 diffractometer. The associated structural parameters—crystallite size (D), lattice strain (ε), and interplanar spacing (d)—were calculated using HighScore Plus© software (version 5.3) with the following equations:

(1) $$D=\left[\frac{\left(k\right)\times (\lambda )}{\left(\beta_{D}\right)\times (Cos\theta )}\right]$$

(2) $$\varepsilon =\left\{\frac{\beta_{D}}{4\times sin\theta }\right\}$$

(3) $$d=\frac{\lambda }{2\sin\theta }$$

Fourier Transform Infrared (FT-IR) spectra were recorded on a Bruker Vertex 70 (Bruker, Germany) (4000–400 cm^−1^, 3 cm^−1^ resolution). Morphology and particle size were examined by field-emission scanning electron microscopy (FE-SEM) using a Quattro S (Thermo Scientific) instrument (Thermo Fisher Scientific Co LLC, Waltham, MA, USA), with elemental composition determined by energy-dispersive X-ray spectroscopy (EDX) (Thermo Fisher Scientific Co LLC, Waltham, MA, USA). Surface chemistry and oxidation states of the final calcined sample (400 °C) were probed via X-ray photoelectron spectroscopy (XPS; Thermo Scientific K-Alpha system (Thermo Fisher Scientific Co LLC, Waltham, MA, USA), Al Kα monochromatic source. Zeta potential and hydrodynamic size measurements were obtained using a Malvern Zetasizer (Malvern Panalytical, Westborough, MA, USA). Thermogravimetric Analysis and Differential Thermal Analysis (TGA & DTA) of Fe-P were measured on a PerkinElmer thermogravimetric analyzer (PerkinElmer, Shelton, CT, USA) in air, with the temperature ramped to 1000 °C at a rate of 10 °C·min^−1^.

### 2.4. Antimicrobial Testing

The antibacterial performance of carbon-doped Fe_2_O_3_ nanocomposite powder was assessed by agar well diffusion against *Escherichia coli* (Gram-negative) and *Staphylococcus aureus* (Gram-positive), employing a standardized inoculum (≈1.5 × 10^8^ CFU·mL^−1^, 0.5 McFarland) and DMSO dispersions (20 and 30 mg·mL^−1^ nominated as C/20 and C/30, respectively) in accordance with CLSI/Kirby–Bauer protocols [18,19,20].

### 2.5. Adsorption Experiments

A stock solution of lead was prepared by dissolving Pb(NO_3_)_3_·6H_2_O (Sigma Aldrich, Darmstadt, Germany) in distilled water. Experiments were carried out in 50 mL Erlenmeyer flasks, by mixing 20 mL of a 10 mg/L lead solution with 0.1 g of Fe_2_O_3_/C microspheres. To assess the effect of pH on lead removal, solutions with pH values ranging from 2.0 to 6.0 were prepared using 0.1 M HCl or NaOH For the kinetic studies, the contact time was varied from 1 to 120 min while keeping other parameters constant. Adsorption isotherms were investigated using initial lead concentrations ranging from 10 to 100 mg/L, with all other experimental conditions held constant. All experiments were performed in triplicate. Lead concentrations in experimental solutions were quantified using Inductively Coupled Plasma Optical Emission Spectroscopy (ICP-OES) (Plasma Quant PQ 9000 Elite, Analytik Jena, Germany).

The adsorption capacity (q) and lead removal efficiency were calculated using Equations (4) and (5):

(4) $$q=\frac{V(C_{i}-C_{f})}{m}$$

(5) $$E=\frac{C_{i}-C_{f}}{C_{i}}\times 100$$

where q is the content of lead adsorbed, mg/g; V is the volume of solution, mL; C_i_ and C_f_ are initial and final lead concentrations in the solution, mg/L; and m is sorbent dosage, g.

## 3. Results and Discussion

### 3.1. Fe_2_O_3_/C Composite Characterization

#### 3.1.1. X-Ray Powder Diffraction Analysis

**Figure 1:**
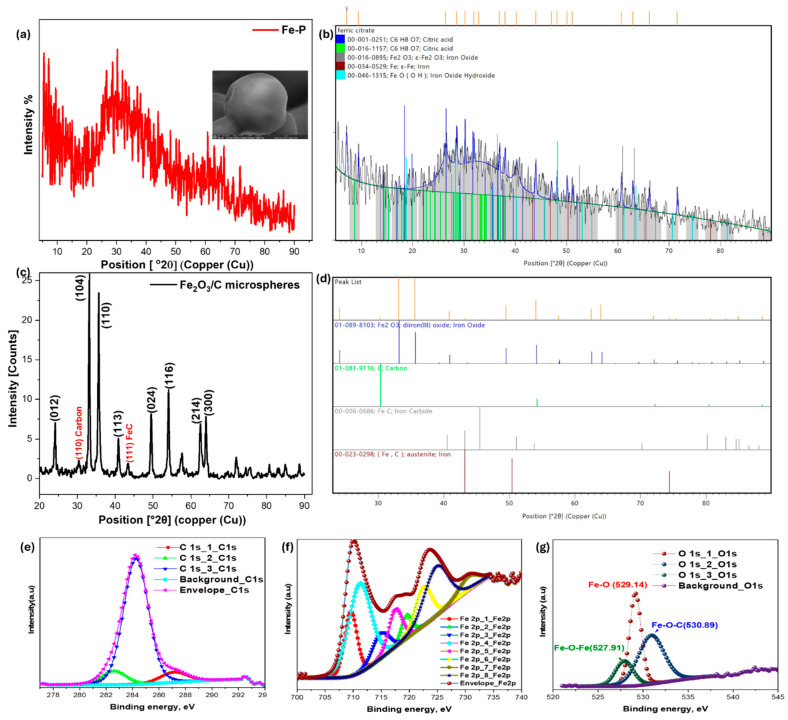
(**a**) Powder X-ray diffraction pattern (PXRD) of the iron–citrate precursor (Fe-P), (**b**) Background-smoothed and profile-refined pattern processed in HighScore Plus^®^, (**c**) PXRD of Iron Oxide doped carbon (Fe_2_O_3_/C) microsphere and (**d**) Extracted peak list with phase indexing and comparison to reference PDF cards for Fe_2_O_3,_ Carbon and relevant iron carbide phases aligned with XPS of (**e**) Carbon, (**f**) Fe and (**g**) Oxygen on nanocomposite surface.

Figure 1a,b show the powder X-ray diffraction pattern of the iron–citrate precursor (Fe-P), which displays a mixed semicrystalline character. A broad, low-intensity background consistent with an amorphous/organic matrix is superimposed with a series of sharp Bragg reflections, indicating retained long-range order in portions of the citrate lattice [21]. The prominent reflections occur at 2θ = 7.152, 9.436, 26.440, 28.573, 30.223, 31.934, 32.873, 36.929, 38.024, 40.249, 43.994, 47.057, 48.144, 50.099, 51.116, 60.704, 62.948, 66.143 and 71.595°, corresponding to d-spacings of 12.349, 9.366, 3.368, 3.122, 2.955, 2.800, 2.722, 2.432, 2.365, 2.239, 2.057, 1.930, 1.889, 1.819, 1.785, 1.524, 1.475 and 1.412 Å, respectively. Most of these reflections match the characteristic planes of crystalline citric acid (JCPDS card 00-001-0251) (PDF S1), confirming the preservation of the citrate framework in the solid precursor. However, systematic small shifts in peak positions and subtle changes in relative intensities are observed, which we attribute to incorporation of Fe^3+^ into the citrate lattice and to formation of localized Fe–O coordination [22,23].

Furthermore, Figure 1b shows additional peaks corresponding to iron-oxide/iron-oxyhydroxide motifs (indexed against references 00-016-0895 and 00-046-1315), indicating incipient ordering of Fe-O sublattices within the organic matrix. After calcination (Figure 1c) the product was identified as phase-pure hematite (α-Fe_2_O_3_)/C composite. The diffraction pattern exhibits characteristic peaks at 2θ = 24.2°, 33.2°, 35.6°, 40.9°, 49.5°, 54.1°, 62.4°, and 64°, which can be indexed to the (012), (104), (110), (113), (024), (116), (214), and (300) crystallographic planes, respectively, was identified. This pattern is consistent with a hexagonal crystal system (space group R-3c, No. 167) and matches the reference pattern for hematite (ICDD PDF #01-084-0306) [24]. Additionally, a distinct peak at 2θ = 30.3° (d = 2.94 Å) is attributed to the (110) plane of carbon (JCPDS 01-081-9116; PDF S2), while the peak at 2θ = 43.4° (d = 2.08 Å) corresponds to austenitic Fe–C (C_0.05_ Fe_0.95_, (JCPDS 00-023-0298), confirming successful incorporation of carbon into the composite. Figure 1d highlights the absence of secondary phases, confirming the high purity of the hematite/carbon composite. Table 1 presents a detailed analysis of the diffraction plane parameters for Fe_2_O_3_/C composite. The XRD results indicate an average crystallite size of approximately 19.4 nm, with an average micro-strain of 0.5%, which reflect a high degree of structural stability and rigidity.

XPS analysis was performed to elucidate the surface chemistry of the Fe_2_O_3_/C composite. In the C 1s spectrum (Figure 1e) the dominant peak at 284.2 eV is attributed to adventitious carbon, with a minor contribution from oxidized-carbon at 287.1 eV. A low-binding-energy shoulder at 282.5 eV is consistent with partial Fe–C interaction, likely at lattice defects [25,26]. The Fe 2p region (Figure 1f) confirms Fe^3+^ as the predominant state, characteristic of α-Fe_2_O_3_. The core-level peaks for Fe 2p_3_/_2_ and Fe 2p_1_/_2_ are observed at 711.4 and 725.0 eV, respectively, with a spin–orbit splitting of ≈13.6 eV. The presence of shake-up satellites further confirms high-spin Fe^3+^ in an octahedral coordination [27,28,29].

A weak component in Fe 2p_3_/_2_ at 709.7 eV indicates a minor Fe^2+^ fraction, likely generated by during calcination and associated with the formation of oxygen vacancies. The O 1s spectrum (Figure 1g) can be deconvoluted into lattice oxygen at 529.1 eV, surface hydroxyl/adsorbed oxygen at 530.8 eV, and a low-energy contribution at 527.9 eV attributable to defect-related oxygen species.

#### 3.1.2. Fourier Transform Infrared Analysis

**Figure 2:**
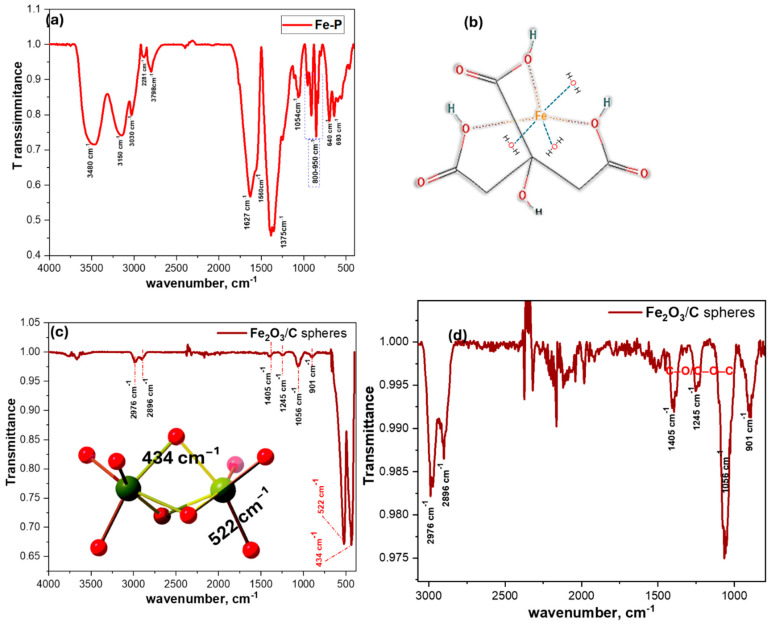
FT-IR spectra of (**a**) iron precursor (Fe-P), (**b**) proposed Fe-P structure, (**c**) hematite microspheres (Fe_2_O_3_/C), and (**d**) magnified region highlighting carbon functionalization on the microsphere surface.

Figure 2a presents the FT-IR spectrum of Fe-P precursor, confirming an oxygen-coordinated, hydrogen-bonded structure. The broad envelope at ~3480 cm^−1^ is assigned to O–H stretching vibrations from coordinated/lattice water and citrate hydroxyl groups, with a minor isolated O–H feature at ~3798 cm^−1^ [22]. The strong asymmetric carboxylate stretch, ν_(COO_^−^_)_ is resolved at 1627 cm^−1^ with a shoulder at 1560 cm^−1^, while the symmetric stretch ν_(COO_^−^_)_ appears at ~1375 cm^−1^. The Δν _(COO_^−^_)_ value of ~252 cm^−1^ suggests mixed carboxylate coordination modes [30], with components consistent with both monodentate/bridging and bidentate/chelating binding. Bands at 1054 cm^−1^ and in the 800–950 cm^−1^ region are attributed to C–O and C–C–O vibrations [31,32,33], while features at ~693 and 640 cm^−1^ correspond to Fe–O stretching/deformation modes. The spectral data, supported by CHN analysis, are consistent with an octahedral FeO_6_ environment in which citrate acts as a tridentate chelate [23], with additional carboxylate bridging and water coordination [34], suggesting structure in Figure 1b.

Following calcination (400 °C, 2 h), the product (Figure 2c) exhibits a spectrum dominated by crystalline hematite (α-Fe_2_O_3_), evidenced by intense Fe–O vibrations at 522 and 434 cm^−1^ [35]. The absence of the precursor’s broad O–H envelope confirms dehydroxylation and dehydration. Weak residual absorptions at 2976, 2896 (C–H), and 1405, 1245, 1056 cm^−1^ (C–O/C–O–C) signify a minor surface carbon phase derived from partial citrate decomposition (Figure 1d) [36]. The spectral evolution may indicate the formation of a carbon-decorated hematite matrix.

#### 3.1.3. Thermal Gravimetric Analysis

**Figure 3:**
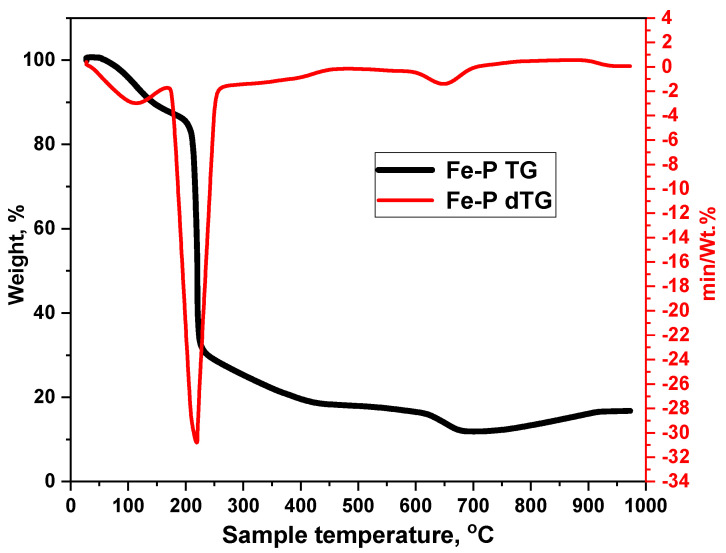
Thermal gravimetric analysis (Fe-P TG) and Differential Thermal Analysis (Fe-P dTG) profiles of the precursor (Fe-P).

Figure 3 shows the TGA and DTG curves of the Fe-P precursor (recorded at 10 °C min^−1^ in air), indicating a three-stage thermal evolution that leads directly to the formation of a carbon-decorated Fe_2_O_3_ matrix. The initial mass loss below ≈150 °C is attributable to removal of crystallization water (dehydration/dehydroxylation) [22]. The dominant, sharp DTG maximum at ≈210–220 °C corresponds to rapid oxidative decomposition and combustion of the citrate ligand, during which the bulk of the organic mass is volatilized as CO_2_/CO and low-molecular fragments [37]. A much slower, minor mass change extending from ≈300 to 700 °C reflects progressive oxidation and structural reorganization of residual carbonaceous fragments and concurrent crystallization of the iron oxide network. The final residue (~16–20 wt%) is commensurate with the theoretical iron-oxide yield and confirms near-quantitative conversion of Fe-citrate to an Fe-oxide phase.

Crucially, the incomplete removal of carbonaceous species during the main combustion step followed by their gradual oxidation at higher temperatures provides a plausible route for a thin, oxygenated carbon layer that remains surface-bound or becomes incorporated as defect carbon within the growing hematite lattice. In other words, the observed thermal behavior is fully consistent with the formation of carbon-doped or carbon-decorated Fe_2_O_3_, rather than a pure oxide product. This residual carbon can rationalize for the weak C–O/C–H features observed in post-calcination FT-IR spectrum and the minor carbon-related signal in the XRD data. It also explains how the thermal decomposition of a citrate precursor yields a composite material in which a predominantly crystalline hematite matrix is modified by trace carbon.

#### 3.1.4. Field Emission Scanning Electron Microscope and Energy Dispersive X-Ray Analysis

**Figure 4:**
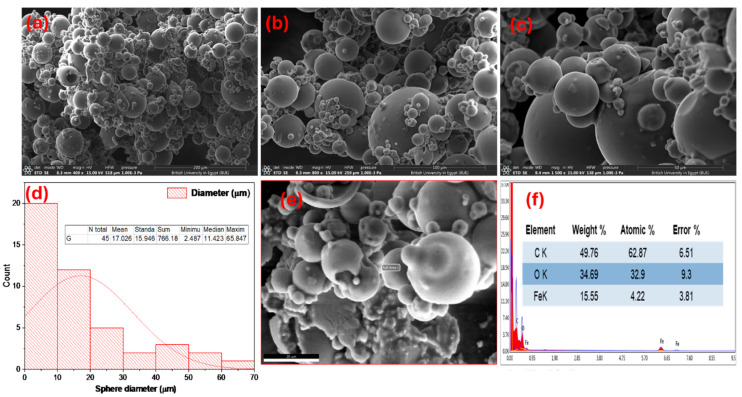
Multimodal characterization of Fe-P spheres, (**a**–**c**) FE-SEM images with various magnification, (**d**) Particles size distribution and (**e**,**f**) Frame EDX mapping analysis.

Figure 4a–c reveal a highly ordered, hierarchical particulate architecture dominated by spherical morphologies with a broad, multimodal size distribution. The population is polydisperse, comprising large primary spheres that act as substrates for numerous smaller secondary and tertiary spheres and particulates adhered to their surfaces.

Main part of primary particles exhibits smooth, near-continuous outer shells, while occasional ruptured or collapsed particles expose hollow interiors, indicating a core–shell or hollow-sphere growth pathway rather than solid, dense beads. Surface details at higher magnification shows nanoscale roughness and numerous sub-100 nm satellite particles decorating the larger spheres within average 17 µm (Figure 4d). These satellites form clusters and partial coatings that would increase external surface area and imply a self-assembly process in which organic constituents (citrate complexes) solidify and agglomerate before complete densification.

The observed morphology is consistent with an organic-templated assembly mechanism. Citrate ligands complex iron ions and promote gel/paste formation during the wet grinding and aging steps. Subsequent solvent removal, condensation and gas evolution then produce spherical shells and vesicle-like voids. The intimate mixture of large and small spheres with the presence of surface pits and thin shell walls suggest that the precursor network is mechanically fragile and rich in volatile and organic components. These components decompose during thermal treatment to yield the final porous oxide frameworks.

Figure 4e,f provide a complementary, semi-quantitative chemical analysis that is fully consistent with an organic-rich iron citrate precursor. The measured weight percentages are C 49.8 wt%, O 34.7 wt% and Fe 15.6 wt%, which translate to atomic percentages of ~62.9% C, 32.9% O and 4.2% Fe. The strong carbon signal and correspondingly high atomic C/Fe ratio may indicate that the particle surfaces and near-surface volumes are dominated by citrate-derived organic material. Conversely, the lower atomic fraction of Fe-despite a non-negligible mass fraction reflects the higher atomic mass of iron and implies that Fe is present as a heavy, but relatively sparse, component in the near-surface region sampled by EDX.

The high residual carbon content and well-defined hollow architecture suggest a formation pathway where Fe(III) is embedded within an organic citrate scaffold that constitutes the shell of the precursor spheres. In solution, ferric citrate trihydrate [Fe(C_6_H_7_O_7_)·3H_2_O] is expected to form polynuclear complexes because citrate, bearing three carboxylates and one hydroxyl group, can chelate a given Fe^3+^ center while simultaneously bridging adjacent Fe^3+^ ions via μ-carboxylate and μ-O/μ-OH linkages within a hydrogen-bonded network of coordinated and lattice water. Progressive solvent removal and mild heating then drive aggregation and cross-linking of these iron–citrate clusters into low-energy, quasi-spherical assemblies. FT-IR signatures such as shifts in ν(COO^−^) and ν(C–O) relative to free citrate and broad O–H bands support the presence of such coordinated, hydrated iron–citrate frameworks. Upon calcination, this hybrid shell converts into α-Fe_2_O_3_ while the citrate matrix partially carbonizes, leaving a thin carbon-rich layer and Fe–O–C/Fe–C motifs distributed through the shell. This process transfers the hollow morphology of the precursor to the final Fe_2_O_3_/C microspheres, as illustrated in Schematic Movie S1 (see Supplementary Materials).

**Figure 5:**
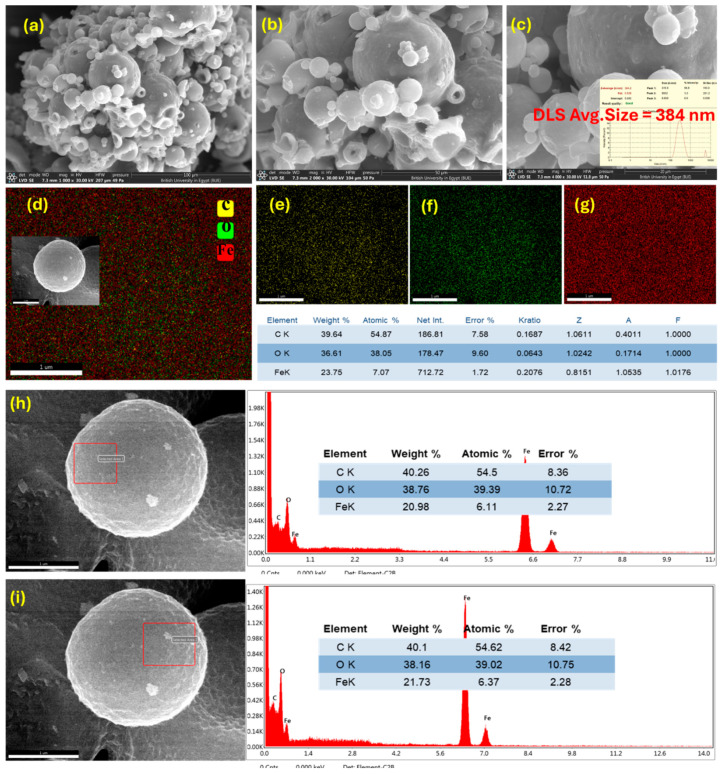
Multimodal characterization of Fe_2_O_3_/C hollow spheres: (**a**–**c**) FE-SEM images at increasing magnifications; (**d**) combined elemental mapping of C, O, and Fe; (**e**–**g**) individual maps of C, O, and Fe, respectively; and (**h**,**i**) localized EDX spectra from distinct surface regions of a representative microsphere.

FE-SEM of the calcined Fe_2_O_3_/C composite (Figure 5) reveals a robust hierarchical architecture comprising well-defined spherical and hollow microstructures with a highly porous shell. At low magnification (Figure 5a), the material displays densely packed microspheres forming agglomerated clusters. Higher magnifications (Figure 5b,c) reveal intricate surface topography characterized by roughened shells and multiple nano- to microscale protrusions. The average particle size is 0.38 µm, with a zeta potential of +15.9 mV, as determined by dynamic light scattering (PDF S3 and S4, respectively). These features are indicative of structural reorganization during calcination and partial gas evolution. The presence of hollow interiors and fractured surfaces suggests that decomposition of the citrate precursor and removal of volatile components generated internal porosity, while localized shell collapse confirms thin-wall formation during organic burnout. Such structural features are advantageous for adsorption and catalytic applications, as they enhance surface area and diffusion pathways. Figure S1 shows cross-sections of the hollow microspheres, revealing an internal void of ~5.3 μm and a shell thickness of ~1.5 μm, indicative of a robust and rigid architecture.

Elemental mapping (Figure 5d–g) provides compelling evidence of a homogeneously distributed Fe–O–C network. Carbon (yellow, Figure 5e), oxygen (green, Figure 5f), and iron (red, Figure 5g) signals are uniformly dispersed across the microspheres without detectable phase segregation, confirming successful incorporation of carbon residues into the iron oxide matrix rather than forming isolated carbonaceous domains. This uniform distribution suggests that carbon persists either as dopant within Fe_2_O_3_ lattice or as a thin, amorphous layers coating the oxide framework, which can facilitate electron transfer and enhance structural stability [38].

Quantitative EDS analysis supports this interpretation, showing weight percentages of C (39.64%), O (36.61%), and Fe (23.75%), corresponding to atomic ratios of ~54.9% C, 38.1% O, and 7.1% Fe. The relatively high surface carbon content significantly higher than the stoichiometric expectation for hematite confirms that residual carbonaceous species remain after calcination, likely derived from incomplete decomposition of citrate ligands and subsequent carbonization at elevated temperatures. These observations indicate that calcination transformed the precursor into a Fe_2_O_3_/C composite with a hierarchical hollow-sphere morphology, high porosity, and carbon doping. The retained carbon not only prevents particle sintering during thermal treatment, preserves the hollow spherical structure, and enhances surface conductivity and provides abundant active sites. The homogeneous elemental distribution reflects a successful synthetic strategy yielding a structurally stable, carbon-functionalized hematite system.

The EDX spectra (Figure 5h,i), collected from two distinct surface regions of the Fe_2_O_3_/C microsphere confirm the coexistence of Fe, O, and C, consistent with carbon-doped hematite formation. Both areas exhibit comparable elemental ratios (C ~40 wt%, O ~38 wt%, Fe ~21 wt%), indicating uniform carbon incorporation across the surface. The high carbon content suggests an intimate Fe–C interface, promoting structural stability and electronic conductivity, slight variations in Fe and O distributions reflect localized surface heterogeneity without phase segregation.

#### 3.1.5. Antimicrobial Activity

**Figure 6:**
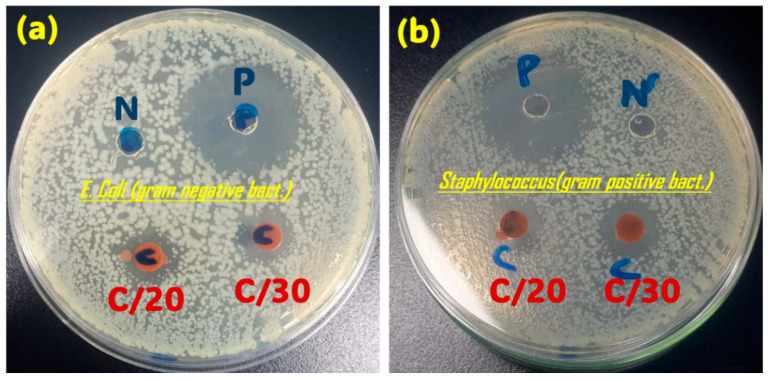
Agar well diffusion test against (**a**) *Escherichia coli* (Gram-negative) and (**b**) *Staphylococcus aureus* (Gram-positive).

Using chloramphenicol as a qualitative reference, the Fe_2_O_3_/C dispersions at 20 and 30 mg·mL^−1^ produced inhibition zones corresponding to roughly 35–41% of the chloramphenicol zone diameter against *E. coli* and 27–36% against *S. aureus,* respectively (Figure 6), This indicates that the nanocomposite exhibits clear antibacterial effects against both Gram-negative and Gram-positive strains. These agar-well diffusion tests were designed as a preliminary screening rather than a comprehensive quantitative microbiological study. Consequently, the observed inhibition halos are interpreted as evidence of intrinsic antibacterial activity rather than a statistically benchmarked measure of potency. The observed antibacterial effects can be rationalized by the cooperative by a cooperative mechanism, Fe_2_O_3_ domains are known to generate reactive oxygen species and perturb bacterial membranes, while the carbonaceous matrix promotes bacterial adhesion and facilitates interfacial electron transfer. This synergy suggests that the Fe_2_O_3_/C nanocomposite is a promising dual-functional component for integrated water purification systems.

#### 3.1.6. Optical Analysis

**Figure 7:**
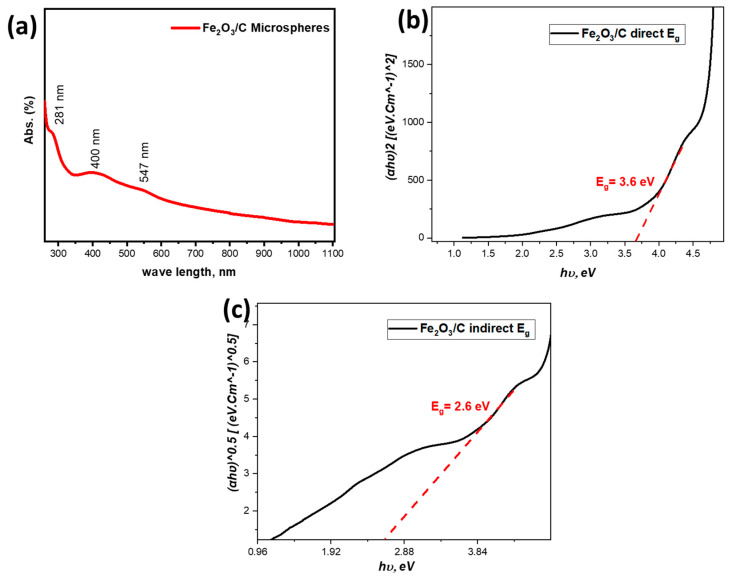
Optical response of the Fe_2_O_3_/C hollow-sphere composite: (**a**) UV–vis spectrum, (**b**) Tauc plot for direct-allowed transitions, (**c**) Tauc plot for indirect-allowed transitions.

The UV-Vis spectrum in Figure 7 shows an intense peak at 281 nm, attributable to π–π* transitions within disordered carbon domains and/or ligand-to-metal charge transfer (O → Fe^3+^). The absorption edge at ~400 nm signifies the onset of interband excitation in the iron oxide phase, while the distinct feature at 547 nm is characteristic of Fe^3+^ d–d and double-excitation transitions in hematite (α-Fe_2_O_3_) [39]. The derived Tauc band gaps are approximately 3.6 eV for the direct transition and 2.6 eV for the indirect transition. The indirect gap of 2.6 eV is moderately higher than the typical bulk hematite value (2.0–2.3 eV), likely due to bigger-size effects, lattice strain, or a defective surface layer. In contrast, the direct gap of 3.6 eV is substantially enlarged and influenced by scattering from the hollow-sphere morphology. Furthermore, the intimate carbon matrix complicates optical analysis by introducing strong UV absorptions that can mask the iron oxide’s intrinsic profile and alter dielectric screening at the interface. This can potentially shift absorption edges and generate new interfacial charge-transfer states [40].

### 3.2. Adsorption Capacity of Fe_2_O_3_/C for Lead Ions

#### 3.2.1. Effect of Experimental Parameters on Lead Adsorption (pH, Time and Lead Concentration)

**Figure 8:**
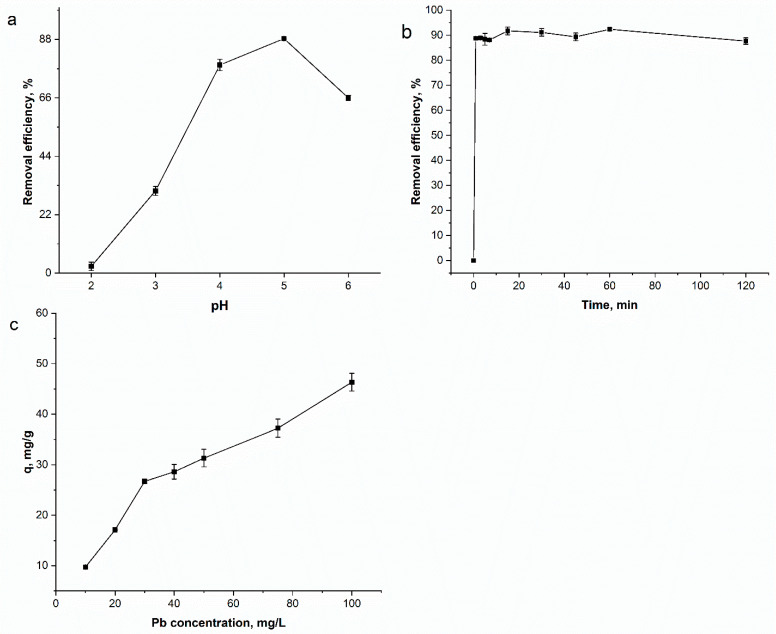
Effect of (**a**) pH, (**b**) contact time and (**c**) lead concentration on lead removal by Fe_2_O_3_/C composite.

The pH plays a decisive role in the removal of metal ions from wastewater, as it affects the surface charge of the adsorbent, the degree of ionization, and the chemical speciation of the metal ions [41]. Experiments were performed within a pH range of 2.0 to 6.0 (Figure 8a) to avoid lead precipitation as a hydroxide, which occurs at higher pH values and would reduce the adsorption rate [42]. Within this range, lead exists primarily as Pb^2+^ and PbOH^+^ [43].

The lowest removal efficiency of 2.6% was attained at pH 2.0. This can be explained by the competition between lead cations and H^+^ ions for adsorption sites [41]. As the pH increased to 5.0, the removal efficiency rose sharply to a maximum of 88% (9.1 mg/g). However, a further increase to pH 6.0 caused a decline in adsorption to 66%. This decline can be associated with the increasing concentration of Na^+^ ions in the solution (from NaOH used for pH adjustment), which compete with lead for exchangeable sites [42]. Consequently, pH 5.0 was selected as the optimal value for all subsequent experiments, a finding consistent with other adsorbents [43,44,45].

The effect of contact time is another crucial parameter for practical application. According to results presented in Figure 8b, lead removal increased rapidly with time until reaching a plateau within 15 min. The initial fast metal adsorption is due to a large number of the available binding sites, while the subsequent equilibrium indicates the saturation of these sites [42,45]. At equilibrium, 92% (9.7 mg/g) of lead ions were removed from the solution.

Finally, the initial lead concentration significantly impacted the adsorption capacity (Figure 8c). The adsorption capacity of Fe_2_O_3_/C composite increased from 9.7 mg/g at 10 mg/L to 46.3 mg/g at 100 mg/L. This increase in the adsorption capacity can be ascribed to the enhanced driving force for mass transfer due to the higher concentration gradient between the solution and the adsorbent surface [44]. Conversely, the percentage removal of lead decreased from 92% to 43.7% over the same concentration range. This occurs because, while the initial number of ions increases, the number of available sorption sites on the adsorbent is fixed. Consequently, the sites become saturated, leading to a lower overall removal percentage [45].

#### 3.2.2. Kinetics and Equilibrium Studies

Adsorption kinetics describe the adsorption rate of the solute at the sorbent–sorbate interface and provide critical insight into the pathways and mechanisms of the adsorption process. The kinetic of lead adsorption onto the Fe_2_O_3_/C composite was analyzed by using two kinetic models: the pseudo-first-order (PFO) and pseudo-second-order (PSO) models.

The pseudo-first-order model (PFO):

(6) $$q_{t}=q_{e}(1-e^{-k_{1}t})$$

The pseudo-second-order model (PSO):

(7) $$q=\frac{q_{e}^{2}k_{2}t}{1+q_{e}k_{2}t}$$

where q_t_ is the amount of lead adsorbed (mg/g) at time t, (mg/g); k_1_ (1/min) and k_2_ (g/mg·min) are the pseudo-first-order and the second-order reaction rate equilibrium constants.

**Figure 9:**
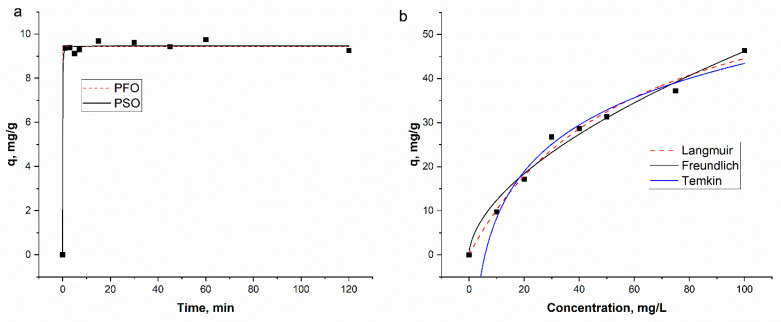
(**a**) non-linear fitting plots of kinetic models; (**b**) non-linear fitting plots of equilibrium models.

Results for the two different kinetic models are given in Figure 9a, and the estimated constants and parameters are reported in Table 2.

The non-linear fitting plots for these models are presented in Figure 9a, and the estimated constants and parameters are reported in Table 2.

The experimentally obtained q_e_ (9.6 mg/g) was in excellent agreement with the values calculated by both the PFO (9.4 mg/g) and PSO (9.5 mg/g) models. According to the coefficient of determination (R^2^) values, both models appeared suitable for explaining the experimental data. To further determine the goodness of fit of the kinetic models, the Akaike information criterion (AIC) test was applied. The AIC results showed that the PFO model provided a better fit for the experimental data. The PSO model is based on the assumption that the rate-controlling step in the sorption process involves chemical interactions (chemisorption) between functional groups of the sorbent and the lead ions [46]. In contrast, the PFO model is often associated with a physisorption-controlled mechanism [47]. The dominance of a PFO mechanism suggests that lead adsorption onto the Fe_2_O_3_/C composite may be primarily physical, though it can also involve interactions with oxygen-containing functional groups such as hydroxyl and carboxylate. Carboxyl group is considered as the most potential functional group on the adsorbents for lead ions binding. High complexation of carboxyl groups with lead ions is explained by lower electrostatic potential compared to hydroxyl groups and the more intense orbital hybridization with Pb atom [48]. Lead removal by acrylate-functionalized hydro-char was reported to be mainly due to the formation of carboxylate-Pb(II) complexes [49]. Multifunctional carboxylic groups also promoted lead adsorption in *Bacillus subtilis* cells [50].

The experimental data were analyzed using Langmuir, Freundlich, and Temkin isotherm models to gain insight into the sorption mechanism and the affinity of the adsorbed ions for the adsorbent (Figure 7b). The calculated adsorption parameters and correlation coefficients obtained from non-linear regression (R^2^) are summarized in Table 2. The Langmuir model suggests a monolayer adsorption and is expressed by the Formula (8):

(8) $$q_{e}=\frac{q_{m\;}bC_{e}}{1+bC_{e}}$$

The mathematical expression of Freundlich isotherm model is presented by Formula (9):

(9) $$q_{e}=K_{F}C^{\frac{1}{n}}$$

The Temkin isotherm model is described by Equation (10):

(10) $$q_{e}=\frac{RT}{b}ln(a_{T}C_{e})$$

where C_e_ is lead ions concentration at equilibrium (mg/L), q_e_ is amount of metal adsorbed at equilibrium (mg/g), q_max_ is maximum adsorption capacity of the sorbent (mg/g) and b is Langmuir adsorption constant (L/mg), K_F_ and n are Freundlich constants that include factors that affect adsorption capacity and adsorption intensity, respectively, 1/b_T_ indicates the sorption potential of the sorbent, and a_T_ is Temkin constant.

Based on the coefficients of determination, the Langmuir isotherm provided the best fit for the adsorption of lead ions onto the FeO_x_/C composite, suggesting a monolayer adsorption process. The Freundlich model, typically used for multilayer adsorption on heterogeneous surfaces under non-ideal conditions [49], yielded a lower R^2^ value. Similarly, the Temkin model, which elucidates the effects of heterogeneous surface interactions on adsorption [49], also demonstrated a poorer fit. The better suitability of the Langmuir model was further confirmed by the AIC test.

The maximum adsorption capacity of Fe_2_O_3_/C composite estimated from Langmuir model was compared with the values reported for lead by other authors (Table 3). The Fe_2_O_3_/C composite demonstrated a higher adsorption capacity than flax fibres, tannin–formaldehyde resin, *Spirulina platensis*, and roasted and grounded barley (*Hordeum vulgare* L.) waste, indicating its superior ability to capture lead ions. It also exhibited comparable performance to citrus limetta leaves, *Schleichera oleosa* bark, steel slag, chemically modified *Opuntia ficus indica Cladodes,* and *Nostoc* sp. MK-11, highlighting its effectiveness as a competitive adsorbent. Honey hydrothermal biochar, acrylate-functionalized hydrochar, and *Enterobacter chuandaensis* DGI-2 exhibited a higher adsorption capacity compared to Fe_2_O_3_/C composite, which can be explained by large number of hydroxyl and carboxylic groups [44].

**Figure 10:**
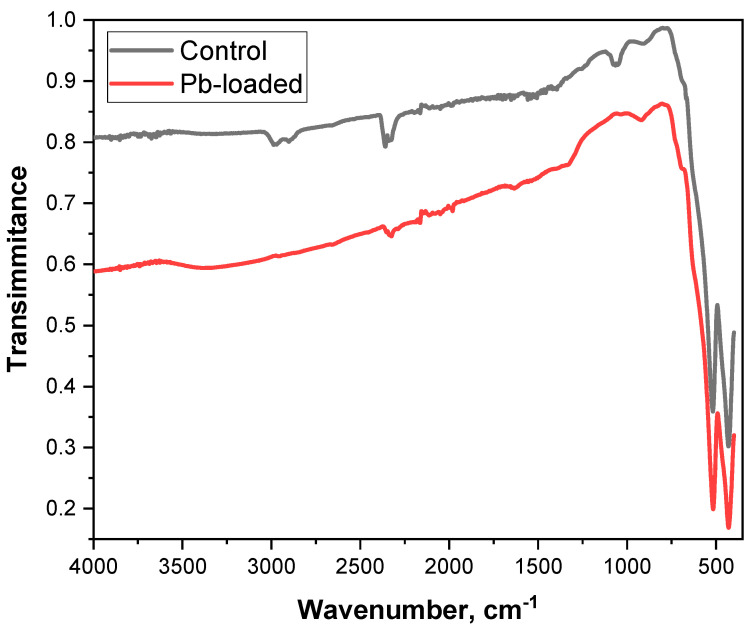
FT-IR spectra of the Fe_2_O_3_/C composite before Pb (II) adsorption (control) and after Pb(II) adsorption (Pb-loaded).

FT-IR spectra of nanocomposite before and after lead adsorption (Figure 10) were recorded to explain mechanism of lead adsorption. In the pristine Fe_2_O_3_/C composite, very weak bands are observed at 2976 and 2896 cm^−1^ (aliphatic C–H stretches) and at 1405, 1245, and 1056 cm^−1^ (C–O/C–O–C vibrations). After lead adsorption, these bands disappear, indicating that the thin, oxygenated carbon layer derived from citrate is not spectroscopically inert but is actively involved in the uptake of metal ions. In the presence of lead, these residual C–O/C–O–C and related groups participate in inner-sphere complexation (–O–Pb, –COO–Pb). These groups may also undergo partial hydrolysis, oxidation, or desorption during the contact with the aqueous solution and subsequent washing steps. Furthermore, the original carbon-related vibrations become attenuated due to surface masking by newly formed Pb–O/Pb–OH species and hydrated inorganic layers. Consequently, the original carbon-related vibrations are either transformed into metal–oxygen/carboxylate modes or reduced below the detection limit of FT-IR, which explains their absence in the spectrum of the Pb-loaded adsorbent.

## 4. Conclusions

This work presents a facile and scalable mechano-thermal method for synthesizing carbon-decorated, hollow, mesoporous α-Fe_2_O_3_ microspheres, which exhibit a sustainable formation mechanism, rapid adsorption kinetics, high capacity for Pb(II) uptake, and intrinsic antibacterial activity. Using inexpensive precursors, FeCl_3_ and citric acid, the resulting material features a hollow architecture with interconnected meso-porosity, nanoscale hematite crystallites, and residual surface carbon, yielding a positively charged surface. This unique structure facilitates fast ion transport into the interior cavity, promotes extensive adsorption and entrapment within the shell, and enables efficient utilization of binding sites. Functionally, the composite achieves adsorption equilibrium within minutes under mild conditions and exhibits a high monolayer adsorption capacity (qₘₐₓ = 70.6 mg·g^−1^). Moreover, the material demonstrates consistent antibacterial activity against common waterborne pathogens, highlighting the potential of its application for complex wastewater treatment: removal of metal ions and pathogens. Overall, these carbon-decorated hollow hematite microspheres represent a practical, low-energy material that meaningfully advances integrated wastewater remediation technology. While this study demonstrates the high efficiency of α-Fe_2_O_3_ microspheres in lead metal removal, the future work should be focused on adsorbent regeneration and reusability, selectivity in complex wastewater matrices, long-term stability and prevention of iron leaching, that can deactivate the material and cause secondary pollution.

## Figures and Tables

**Table 1:** Calculated structural parameters of the Fe_2_O_3_/C composite.

Position, °2θ	d-Spacing Å	Height, cts	Height, cps	Relative Intensity, %	Area, cts × °2θ	Area, cps × °2θ	Crystallite Size Only, Å	Micro Strain Only, %	FWHM Total, °2θ
24.10	3.6893	4.91	10.9	28.63	2.19	4.87	244	0.755	0.216
30.33	2.9439	1.26	2.79	7.34	1.2	2.67	104	1.409	0.463
33.12	2.7025	17.13	38.08	100	7.71	17.14	242	0.557	0.312
35.62	2.5184	16.48	36.63	96.21	8.13	18.07	222	0.566	0.248
40.83	2.20812	3.42	7.59	19.94	2.04	4.54	181	0.611	0.353
43.30	2.0875	0.92	2.05	5.37	0.66	1.46	134	0.779	0.690
49.47	1.8407	5.87	13.05	34.27	2.97	6.6	219	0.419	0.374
54.03	1.6957	7.84	17.43	45.78	4.05	9.01	223	0.379	0.352
57.48	1.6018	1.77	3.94	10.35	2.56	5.68	74	1.081	0.789
62.50	1.4847	4.64	10.31	27.09	3.49	7.75	155	0.479	0.495
63.93	1.4548	6.17	13.7	35.98	2.94	6.54	261	0.278	0.293
71.97	1.3109	1.74	3.88	10.18	1.17	2.61	179	0.366	0.508
74.41	1.2738	0.75	1.67	4.38	0.45	0.99	217	0.293	0.377
75.46	1.2586	0.77	1.72	4.51	0.48	1.07	180	0.350	0.606
80.65	1.1902	0.76	1.69	4.44	0.85	1.89	113	0.528	0.612
83.03	1.1621	0.65	1.44	3.79	0.54	1.19	143	0.406	0.801
84.91	1.1411	1.44	3.2	8.4	0.71	1.58	295	0.193	0.308
88.58	1.1030	1.41	3.14	8.24	0.69	1.53	311	0.177	0.245

**Table 2:** The constants and correlation coefficients of the kinetic and equilibrium models.

**PFO**						**PSO**					
qe		K_1_		R^2^		qe		K_2_		R^2^	
9.4 ± 0.06		6.5 ± 0.07		0.99		9.5 ± 0.086		5.7 ± 7.6		0.99	
**Langmuir**				**Freundlich**				**Temkin**			
q_m_	b		R^2^	K_f_	n		R^2^	a	b		R^2^
70.9 ± 6.6	0.02 ± 0.003		0.99	3.3 ± 0.6	1.7 ± 0.1		0.96	0.17 ± 0.023	156 ± 10.6		0.95

**Table 3:** Comparison of the adsorption capacity of Fe_2_O_3_/C composite with literature data.

Adsorbent	q, mg/g	pH	Reference
Honey hydrothermal biochar	132	5.0	[44]
Flax fibres	10.7	6.0	[42]
Acrylate-functionalized hydrochar	193	5.0	[49]
Tannin–formaldehyde resin	13.8	3.5	[51]
*Spirulina platensis*	4.8	3.0	[43]
*Enterobacter chuandaensis* DGI-2	98.6	6.5	[52]
*Nostoc* sp. MK-11	83.9	4.0	[53]
Chemically Modified *Opuntia ficus indica Cladodes*	64.7	5.0	[54]
Roasted and grounded barley (*Hordeum vulgare* L.) waste	25.76	5.5	[55]
Steel slag	59.8		[56]
Citrus limetta leaves	69.8	6	[57]
*Schleichera oleosa* bark	69.4	6	[58]
Fe_2_O_3_/C composite	70.6	5.0	Present study

## Data Availability

The original contributions presented in this study are included in the article/Supplementary Material. Further inquiries can be directed at the corresponding author.

## Supplementary Materials

The following supporting information can be downloaded at: https://www.mdpi.com/article/10.3390/nano15241850/s1, Figure S1 shows cross-sections of the hollow microspheres, revealing an internal void of ~5.3 μm and a shell thickness of ~1.5 μm, indicative of a robust and rigid architecture.

## References

[1] Diao Z., Zhou Y., Huang K., Alasmary Z. (2025). Lead (Pb) Contamination in Soil and Plants at Military Shooting Ranges and Its Mitigation Strategies: A Comprehensive Review. Processes.

[2] Nadeem M., Mahmood A., Shahid S.A., Shah S.S., Khalid A.M., McKay G. (2006). Sorption of lead from aqueous solution by chemically modified carbon adsorbents. J. Hazard. Mater..

[3] El-wahaab B.A., El-Shwiniy W.H., Alrowais R., Nasef B.M., Said N. (2025). Adsorption of Lead (Pb(II)) from Contaminated Water onto Activated Carbon: Kinetics, Isotherms, Thermodynamics, and Modeling by Artificial Intelligence. Sustainability.

[4] Collin S., Baskar A., Geevarghese D.M., Ali M.N.V.S., Bahubali P., Choudhary R., Lvov V., Tovar G.I., Senatov F., Koppala S. (2022). Bioaccumulation of lead (Pb) and its effects in plants: A review. J. Hazard. Mater. Lett..

[5] Gupta S., Mitra P., Sharma P. (2025). Unmasking Lead Exposure and Neurotoxicity: Epigenetics, Extracellular Vesicles, and the Gut-Brain Connection. Indian J. Clin. Biochem..

[6] Bouida L., Rafatullah M., Kerrouche A., Qutob M., Alosaimi A.M., Alorfi H.S., Hussein M.A. (2022). A Review on Cadmium and Lead Contamination: Sources, Fate, Mechanism, Health Effects and Remediation Methods. Water.

[7] Patra R., Dash P., Panda P.K., Yang P.C. (2023). A Breakthrough in Photocatalytic Wastewater Treatment: The Incredible Potential of g-C3N4/Titanate Perovskite-Based Nanocomposites. Nanomaterials.

[8] Fouda-Mbanga B.G., Onotu O.P., Tywabi-Ngeva Z. (2024). Advantages of the reuse of spent adsorbents and potential applications in environmental remediation: A review. Green Anal. Chem..

[9] Chong W.C., Choo Y.L., Koo C.H., Pang Y.L., Lai S.O. (2019). Adsorptive membranes for heavy metal removal—A mini review. AIP Conf. Proc..

[10] Albqmi M., Frontistis Z., Raji Z., Karim A., Karam A., Khalloufi S. (2023). Adsorption of Heavy Metals: Mechanisms, Kinetics, and Applications of Various Adsorbents in Wastewater Remediation—A Review. Waste.

[11] Hang B.T., Anh T.T. (2021). Controlled synthesis of various Fe_2_O_3_ morphologies as energy storage materials. Sci. Rep..

[12] Wang A., Breakwell C., Foglia F., Tan R., Lovell L., Wei X., Wong T., Meng N., Li H., Seel A. (2024). Selective ion transport through hydrated micropores in polymer membranes. Nature.

[13] Xu L., Xia J., Wang K., Wang L., Li H., Xu H., Huang L., He M. (2013). Ionic liquid assisted synthesis and photocatalytic properties of α-Fe2O3 hollow microspheres. Dalton Trans..

[14] Cao S.W., Zhu Y.J., Microspheres M. (2011). Monodisperse α-Fe_2_O_3_ Mesoporous Microspheres: One-Step NaCl-Assisted Microwave-Solvothermal Preparation, Size Control and Photocatalytic Property. Nanoscale Res. Lett..

[15] Zhang Y., Chu Y., Dong L. (2007). One-step synthesis and properties of urchin-likePS/α-Fe_2_O_3_ composite hollow microspheres. Nanotechnology.

[16] Gomaa I., Alsaiari R.A., Morsy M., Rizk M.A. (2025). Autonomous sampling of α-Fe_2_O_3_ hollow microspheres with carbon-stabilized defects: Calcination-tuned humidity sensor performance. Curr. Appl. Phys..

[17] Hosny N.M., Gomaa I., El-Moemen A.A., Anwar Z.M. (2021). Adsorption of Ponceau Xylidine dye by synthesised Mn_2_O_3_ nanoparticles. Int. J. Environ. Anal. Chem..

[18] Hudzicki J. (2009). Kirby-Bauer Disk Diffusion Susceptibility Test Protocol. Am. Soc. Microbiol..

[19] CDC (2020). Selective Reporting of Antimicrobial Susceptibility Testing Results: A Primer for Antibiotic Stewardship Programs.

[20] (2016). Methods for Antimicrobial Dilution and Disk Susceptibility Testing of Infrequently Isolated or Fastidious Bacteria.

[21] Dudenko D.V., Williams P.A., Hughes C.E., Antzutkin O.N., Velaga S.P., Brown S.P., Harris K.D.M. (2013). Exploiting the synergy of powder x-ray diffraction and solid-state NMR spectroscopy in structure determination of organic molecular solids. J. Phys. Chem. C.

[22] Hosny N.M., Gomaa I., Elmahgary M.G., Ibrahim M.A. (2023). ZnO doped C: Facile synthesis, characterization and photocatalytic degradation of dyes. Sci. Rep..

[23] Ou X., Quan X., Chen S., Zhang F., Zhao Y. (2008). Photocatalytic reaction by Fe(III)–citrate complex and its effect on the photodegradation of atrazine in aqueous solution. J. Photochem. Photobiol. A Chem..

[24] Shinde P.S., Go G.H., Lee W.J. (2012). Facile growth of hierarchical hematite (α-Fe_2_O_3_) nanopetals on FTO by pulse reverse electrodeposition for photoelectrochemical water splitting. J. Mater. Chem..

[25] Rodríguez D.G., Gleeson M.A., Lauritsen J.V., Li Z., Yu X., Niemantsverdriet J.W.H., Weststrate C.J.K.-J. (2022). Iron carbide formation on thin iron films grown on Cu(1 0 0): FCC iron stabilized by a stable surface carbide. Appl. Surf. Sci..

[26] Briggs D. (1981). Handbook of X-ray Photoelectron Spectroscopy C. D. Wanger, W. M. Riggs, L. E. Davis, J. F. Moulder and G. E.Muilenberg Perkin-Elmer Corp., Physical Electronics Division, Eden Prairie, Minnesota, USA, 1979. 190 pp. $195. Surf. Interface Anal..

[27] Yamashita T., Hayes P. (2008). Analysis of XPS spectra of Fe^2+^ and Fe^3+^ ions in oxide materials. Appl. Surf. Sci..

[28] Wu C., Pei Z., Lv M., Huang D., Wang Y., Yuan S. (2023). Polypyrrole-Coated Low-Crystallinity Iron Oxide Grown on Carbon Cloth Enabling Enhanced Electrochemical Supercapacitor Performance. Molecules.

[29] Mills P., Sullivan J.L. (1983). A study of the core level electrons in iron and its three oxides by means of X-ray photoelectron spectroscopy. J. Phys. D Appl. Phys..

[30] Deacon G.B., Phillips R.J. (1980). Relationships between the carbon-oxygen stretching frequencies of carboxylato complexes and the type of carboxylate coordination. Coord. Chem. Rev..

[31] Barraclough C.G., Bradley D.C., Lewis J., Thomas I.M. (1961). 510. The infrared spectra of some metal alkoxides, trialkylsilyloxides, and related silanols. J. Chem. Soc. (Resumed).

[32] Anwaar S., Altaf F., Anwar T., Qureshi H., Siddiqi E.H., Soufan W., Zaman W. (2024). Biogenic synthesis of copper oxide nanoparticles using Eucalyptus globulus Leaf Extract and its impact on germination and Phytochemical composition of Lactuca sativa. Sci. Rep..

[33] Rozali M.L.H., Ahmad Z., Isa M.I.N. (2015). Interaction between Carboxy Methylcellulose and Salicylic Acid Solid Biopolymer Electrolytes. Adv. Mat. Res..

[34] West C.P., Morales A.C., Ryan J., Misovich M.V., Hettiyadura A.P.S., Rivera-Adorno F., Tomlin J.M., Darmody A., Linn B.N., Lin P. (2023). Molecular investigation of the multi-phase photochemistry of Fe(III)–citrate in aqueous solution. Environ. Sci. Process Impacts.

[35] Basavaraja S., Balaji D.S., Bedre M.D., Raghunandan D., Swamy P.M.P., Venkataraman A. (2011). Solvothermal synthesis and characterization of acicular α-Fe_2_O_3_ nanoparticles. Bull. Mater. Sci..

[36] Max J.J., Chapados C. (2002). Infrared spectroscopy of aqueous carboxylic acids: Malic acid. J. Phys. Chem. A.

[37] Kristl M., Muršec M., Šuštar V., Kristl J. (2016). Application of thermogravimetric analysis for the evaluation of organic and inorganic carbon contents in agricultural soils. J. Therm. Anal. Calorim..

[38] Liu R., Xu S., Shao X., Wen Y., Shi X., Hu J., Yang Z. (2021). Carbon coating on metal oxide materials for electrochemical energy storage. Nanotechnology.

[39] Cao W., Jiang Z., Gai C., Barrón V., Torrent J., Zhong Y., Liu Q. (2022). Re-Visiting the Quantification of Hematite by Diffuse Reflectance Spectroscopy. Minerals.

[40] Hofmeister A.M., Pitman K.M., Goncharov A.F., Speck A.K. (2009). Optical constants of silicon carbide for astrophysical applications. ii. extending optical functions from infrared to ultraviolet using single-crystal absorption spectra. Astrophys. J..

[41] Ghahremani A., Manteghian M., Kazemzadeh H. (2021). Removing lead from aqueous solution by activated carbon nanoparticle impregnated on lightweight expanded clay aggregate. J. Environ. Chem. Eng..

[42] Abbar B., Alem A., Marcotte S., Pantet A., Ahfir N.D., Bizet L., Duriatti D. (2017). Experimental investigation on removal of heavy metals (Cu^2+^, Pb^2+^, and Zn^2+^) from aqueous solution by flax fibres. Process Saf. Environ. Prot..

[43] Zinicovscaia I., Yushin N., Rodlovskaya E., Kamanina I. (2017). Biosorption of lead ions by cyanobacteria Spirulina platensis: Kinetics, equilibrium and thermodynamic study. Nova Biotechnol. Chim..

[44] Wang B., Yu J., Liao H., Zhu W., Ding P., Zhou J. (2020). Adsorption of Lead (II) from Aqueous Solution with High Efficiency by Hydrothermal Biochar Derived from Honey. Int. J. Environ. Res. Public Health.

[45] Cherono F., Mburu N., Kakoi B. (2021). Adsorption of lead, copper and zinc in a multi-metal aqueous solution by waste rubber tires for the design of single batch adsorber. Heliyon.

[46] Bulgariu L., Balan C., Bulgariu D., Macoveanu M. (2014). Valorisation of romanian peat for the removal of some heavy metals from aqueous media. Desalination Water Treat..

[47] Khamwichit A., Dechapanya W., Dechapanya W. (2022). Adsorption kinetics and isotherms of binary metal ion aqueous solution using untreated venus shell. Heliyon.

[48] Zhou X.Y., Wu Y.W., Cai Q., Mi T.G., Zhang B., Zhao L., Lu Q. (2022). Interaction mechanism between lead species and activated carbon in MSW incineration flue gas: Role of different functional groups. Chem. Eng. J..

[49] Yu K., Huan W.W., Teng H.J., Guo J.Z., Li B. (2024). Effect of oxygen-containing functional group contents on sorption of lead ions by acrylate-functionalized hydrochar. Environ. Pollut..

[50] Qu C., Yang S., Mortimer M., Zhang M., Chen J., Wu Y., Chen W., Cai P., Huang Q. (2022). Functional group diversity for the adsorption of lead(Pb) to bacterial cells and extracellular polymeric substances. Environ. Pollut..

[51] Luzardo F.H.M., Velasco F.G., Correia I.K.S., Silva P.M.S., Salay L.C. (2017). Removal of lead ions from water using a resin of mimosa tannin and carbon nanotubes. Environ. Technol. Innov..

[52] Pal P., Ghosh S.K., Mondal S., Maiti T.K. (2025). Lead (Pb^2+^) biosorption and bioaccumulation efficiency of Enterobacter chuandaensis DGI-2: Isotherm, kinetics and mechanistic study for bioremediation. J. Hazard. Mater..

[53] Kaleem M., Minhas L.A., Hashmi M.Z., Ali M.A., Mahmoud R.M., Saqib S., Nazish M., Zaman W., Mumtaz A.S. (2023). Biosorption of Cadmium and Lead by Dry Biomass of *Nostoc* sp. MK-11: Kinetic and Isotherm Study. Molecules.

[54] Lavado-Meza C., Fernandez-Pezua M.C., Gamarra-Gómez F., Sacari-Sacari E., Angeles-Suazo J., Dávalos-Prado J.Z. (2023). Single and Binary Removals of Pb(II) and Cd(II) with Chemically Modified Opuntia ficus indica Cladodes. Molecules.

[55] Feitosa A.G., Santos Y.T.d.C., Menezes J.M.C., Coutinho H.D.M., Teixeira V.F., da Silva J.H., Filho F.J.d.P., Teixeira R.N.P., Oliveira T.M.B.F. (2023). Mielle Brito Ferreira Oliveira, Evaluation of Pb2+ ion adsorption by roasted and grounded barley (*Hordeum vulgare* L.) waste. Results Chem..

[56] Pfeifer A., Škerget M., Čolnik M. (2021). Removal of iron; copper, and lead from aqueous solutions with zeolite, bentonite, and steel slag. Sep. Sci. Technol..

[57] Aboli E., Jafari D., Esmaeili H. (2020). Heavy metal ions (lead, cobalt, and nickel) biosorption from aqueous solution onto activated carbon prepared from Citrus limetta leaves. Carbon. Letters.

[58] Khatoon A., Uddin M.K., Rao R.A.K. (2018). Adsorptive remediation of Pb(II) from aqueous media using Schleichera oleosa bark. Environ. Technol. Innov..

