# Supplementary material for: Synthesis and Characterization of Eco-Engineered Hollow Fe2O3/Carbon Nanocomposite Spheres: Evaluating Structural, Optical, Antibacterial, and Lead Adsorption Properties
Source: Nanomaterials (Basel). 2025 Dec 10;15(24):1850. doi: 10.3390/nano15241850 (PMC12735782; doi:10.3390/nano15241850)
Supplement: Supplementary file 1 [file nanomaterials-15-01850-s001.zip › Figure S1.pdf]

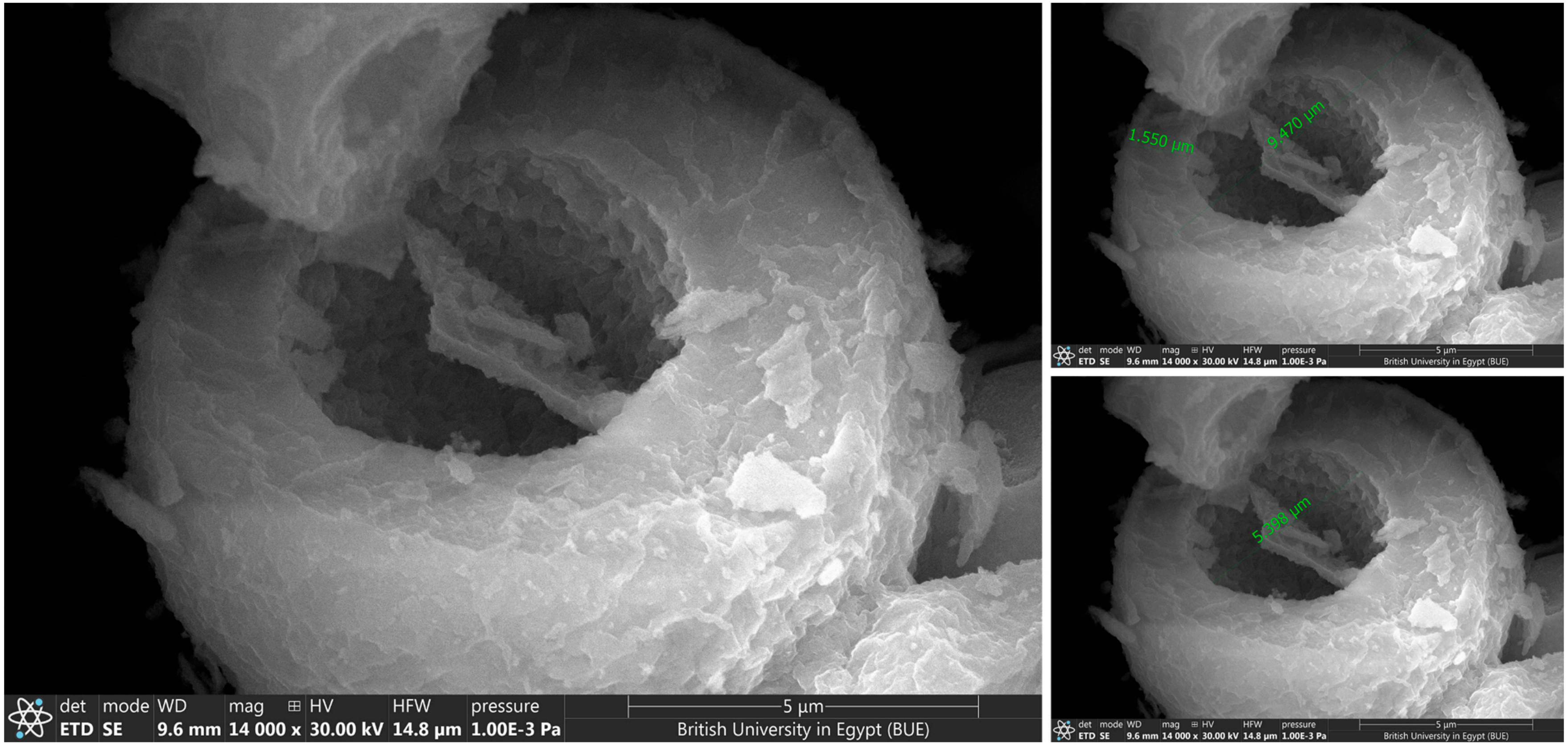

Figure S1 shows cross-sections of the hollow microspheres, revealing an internal void of ~5.3 μm and a shell thickness of ~1.5 μm, indicative of a robust and rigid architecture.
