# Supplementary material for: Synthesis and Characterization of Eco-Engineered Hollow Fe2O3/Carbon Nanocomposite Spheres: Evaluating Structural, Optical, Antibacterial, and Lead Adsorption Properties
Source: Nanomaterials (Basel). 2025 Dec 10;15(24):1850. doi: 10.3390/nano15241850 (PMC12735782; doi:10.3390/nano15241850)
Supplement: Supplementary file 1 [file nanomaterials-15-01850-s001.zip › PDF S1.pdf]

Name and formula

Reference code: 00-001-0251

Compound name: Citric acid

Empirical formula: $C_6H_8O_7$
Chemical formula: $C_6H_8O_7$

Crystallographic parameters

Crystal system: Unknown

RIR:: -

Status, subfiles and quality

Status: Marked as deleted by ICDD
Subfiles: Excipient, Organic, Pharmaceutical
Quality: Low precision (O)

Comments

Creation Date: 9/1/1951
Deleted Or Rejected By: Deleted: Post parcel September 10, 1964; see 00-016-1157. Warning: Unindexed pattern.

References

Primary reference: Hanawalt, J., et al., Anal. Chem., **10**, 475, (1938)

Peak list

No.	h	k	l	d [Å]	2θ [°]	I [%]
1				6.40000	13.826	25.0
2				5.40000	16.402	8.0
3				4.94000	17.942	100.0
4				4.57000	19.408	43.0
5				4.10000	21.658	10.0
6				3.73000	23.836	31.0
7				3.42000	26.033	100.0
8				3.10000	28.776	25.0
9				2.86000	31.249	75.0
10				2.66000	33.666	31.0
11				2.55000	35.165	15.0
12				2.48000	36.191	50.0
13				2.42000	37.121	20.0
14				2.30000	39.134	10.0
15				2.20000	40.991	13.0
16				2.09000	43.254	25.0
17				1.77000	51.596	8.0

Stick Pattern

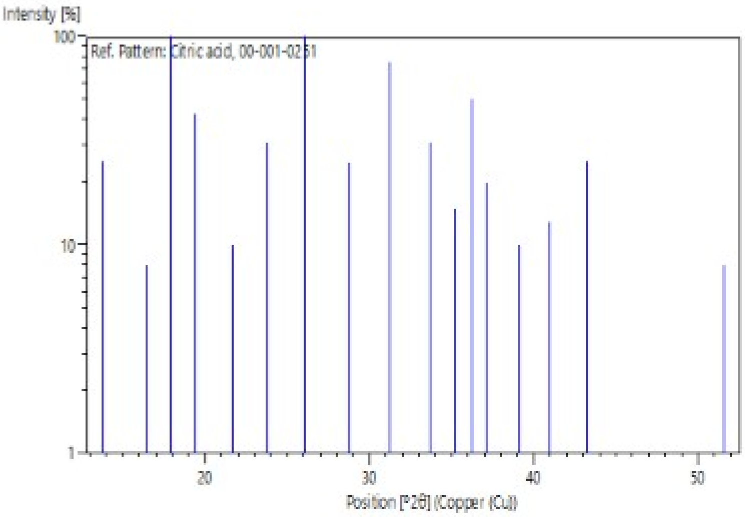
Intensity [%]
100
10
1
Ref. Pattern: Citric acid, 00-001-0251
20
30
40
50
Position [°2θ] (Copper (Cu))
