# Supplementary material for: Synthesis and Characterization of Eco-Engineered Hollow Fe2O3/Carbon Nanocomposite Spheres: Evaluating Structural, Optical, Antibacterial, and Lead Adsorption Properties
Source: Nanomaterials (Basel). 2025 Dec 10;15(24):1850. doi: 10.3390/nano15241850 (PMC12735782; doi:10.3390/nano15241850)

Name and formula

Reference code:	01-081-9116
Compound name:	Carbon
Empirical formula:	C
Chemical formula:	C

Crystallographic parameters

Crystal system:	Cubic
Space group:	I4132
Space group number:	214
a (Å):	4.1160
b (Å):	4.1160
c (Å):	4.1160
Alpha (°):	90.0000
Beta (°):	90.0000
Gamma (°):	90.0000
Volume of cell (10^6 pm^3):	69.73
Z:	8.00
RIR:	2.14

Status, subfiles and quality

Status:	Alternate Pattern
Subfiles:	Alloy, metal or intermetalic, Ceramic, Ceramic - Semiconductor, ICSD Pattern, Inorganic
Quality:	Hypothetical (H)

Comments

ANX:	N
ICSD collection code:	186176
Creation Date:	9/1/2014
Modification Date:	9/1/2022
Cross-References:	ICSD:186176
ANX:	N
Analysis:	C1
Formula from original source:	C
ICSD Collection Code:	186176
Hypothetical Structure:	Structure calculated theoretically
Calculated Pattern Original Remarks:	K4-Carbon, mechanically unstable. DFT calculations using PW-PP method with PBE-GGA and CASTEP code
Minor Warning:	Magnitude of e.s.d. on cell dimension is >1000 ppm
Wyckoff Sequence:	a
Unit Cell Data Source:	Powder Diffraction.

References

Primary reference: Wen Bin, Takami, S., Kawazoe, Y., Adschiri, T., J. Phys. Chem. Solids, **73**, 1264, (2012)
Structure: Wen Bin, Takami, S., Kawazoe, Y., Adschiri, T., J. Phys. Chem. Solids, **73**, 1264, (2012)

Peak list

No.	h	k	l	d [Å]	2θ [°]	I [%]
1	1	1	0	2.91045	30.694	100.0
2	2	1	1	1.68035	54.570	18.3
3	3	1	0	1.30159	72.572	5.1
4	2	2	2	1.18819	80.827	4.5
5	3	2	1	1.10005	88.893	4.8
6	4	0	0	1.02900	96.937	1.8
7	4	1	1	0.97015	105.122	2.3
8	3	3	2	0.87753	122.756	1.2
9	4	3	1	0.80721	145.211	3.4

Stick Pattern

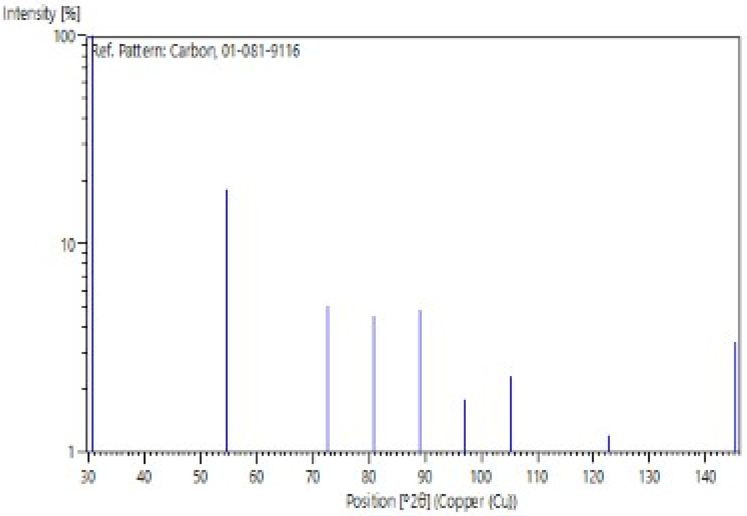

Supplement: Supplementary file 1 [file nanomaterials-15-01850-s001.zip › PDF S2.pdf]
