# Supplementary material for: Synthesis and Characterization of Eco-Engineered Hollow Fe2O3/Carbon Nanocomposite Spheres: Evaluating Structural, Optical, Antibacterial, and Lead Adsorption Properties
Source: Nanomaterials (Basel). 2025 Dec 10;15(24):1850. doi: 10.3390/nano15241850 (PMC12735782; doi:10.3390/nano15241850)

Size Distribution Report by Intensity

v2.2

Malvern

Sample Details

Sample Name: C-Fe2O3 1

SOP Name: mansettings.nano

General Notes:

**File Name:**	Dr. Islam.dts	**Dispersant Name:**	Water
**Record Number:**	362	**Dispersant RI:**	1.330
**Material RI:**	1.59	**Viscosity (cP):**	0.8872
**Material Absorbtion:**	0.010	**Measurement Date and Time:**	Sunday, April 28, 2024 12:46:...

System

**Temperature (°C):**	25.0	**Duration Used (s):**	70
**Count Rate (kcps):**	180.6	**Measurement Position (mm):**	5.50
**Cell Description:**	Clear disposable zeta cell	**Attenuator:**	6

Results

			Size (d.nm):	% Intensity:	St Dev (d.n...
**Z-Average (d.nm):**	384.2	**Peak 1:**	315.0	96.8	110.0
**PdI:**	0.526	**Peak 2:**	5502	3.2	201.2
**Intercept:**	0.882	**Peak 3:**	0.000	0.0	0.000
**Result quality :**	Good				

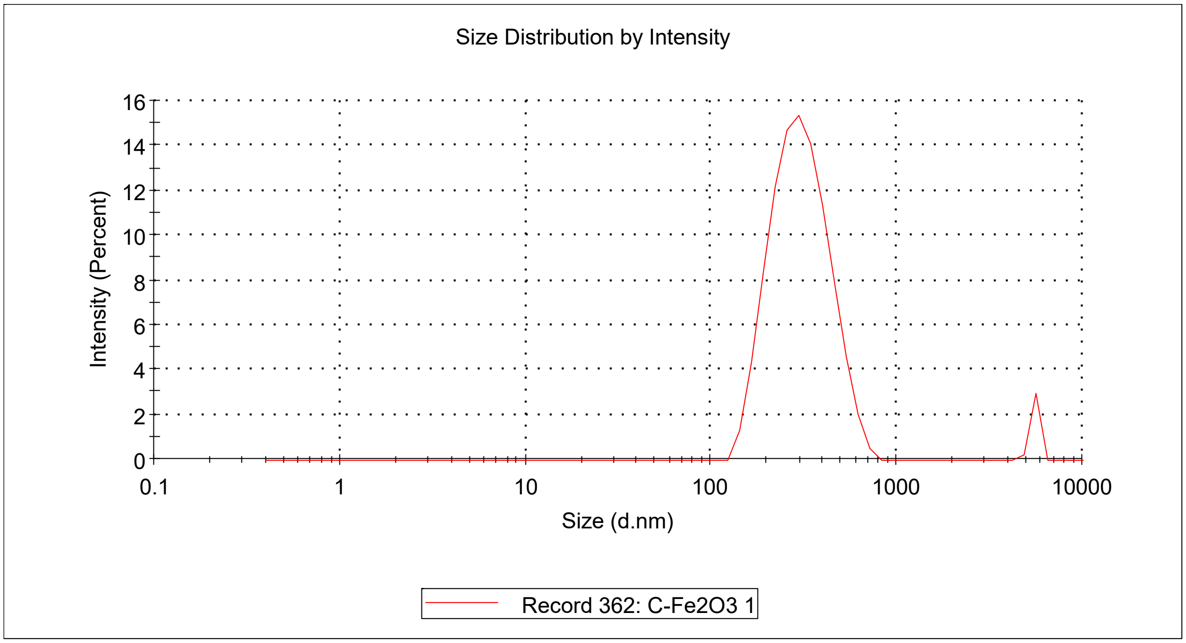

Supplement: Supplementary file 1 [file nanomaterials-15-01850-s001.zip › PDF S3.pdf]
