# Supplementary material for: Synthesis and Characterization of Eco-Engineered Hollow Fe2O3/Carbon Nanocomposite Spheres: Evaluating Structural, Optical, Antibacterial, and Lead Adsorption Properties
Source: Nanomaterials (Basel). 2025 Dec 10;15(24):1850. doi: 10.3390/nano15241850 (PMC12735782; doi:10.3390/nano15241850)

Zeta Potential Report
v2.3

Malvern Instruments Ltd - © Copyright 2008

Sample Details

Sample Name: C-Fe2O3 1

SOP Name: mansettings.nano

General Notes:

**File Name:**	Dr. Islam.dts	**Dispersant Name:**	Water
**Record Number:**	361	**Dispersant RI:**	1.330
**Date and Time:**	Sunday, April 28, 2024 12:37:51 ...	**Viscosity (cP):**	0.8872
		**Dispersant Dielectric Constant:**	78.5

System

**Temperature (°C):**	25.0	**Zeta Runs:**	12
**Count Rate (kcps):**	155.0	**Measurement Position (mm):**	2.00
**Cell Description:**	Clear disposable zeta cell	**Attenuator:**	7

Results

			Mean (mV)	Area (%)	St Dev (mV)
**Zeta Potential (mV):**	15.9	**Peak 1:**	15.9	100.0	4.59
**Zeta Deviation (mV):**	4.59	**Peak 2:**	0.00	0.0	0.00
**Conductivity (mS/cm):**	0.00728	**Peak 3:**	0.00	0.0	0.00
**Result quality :**	Good				

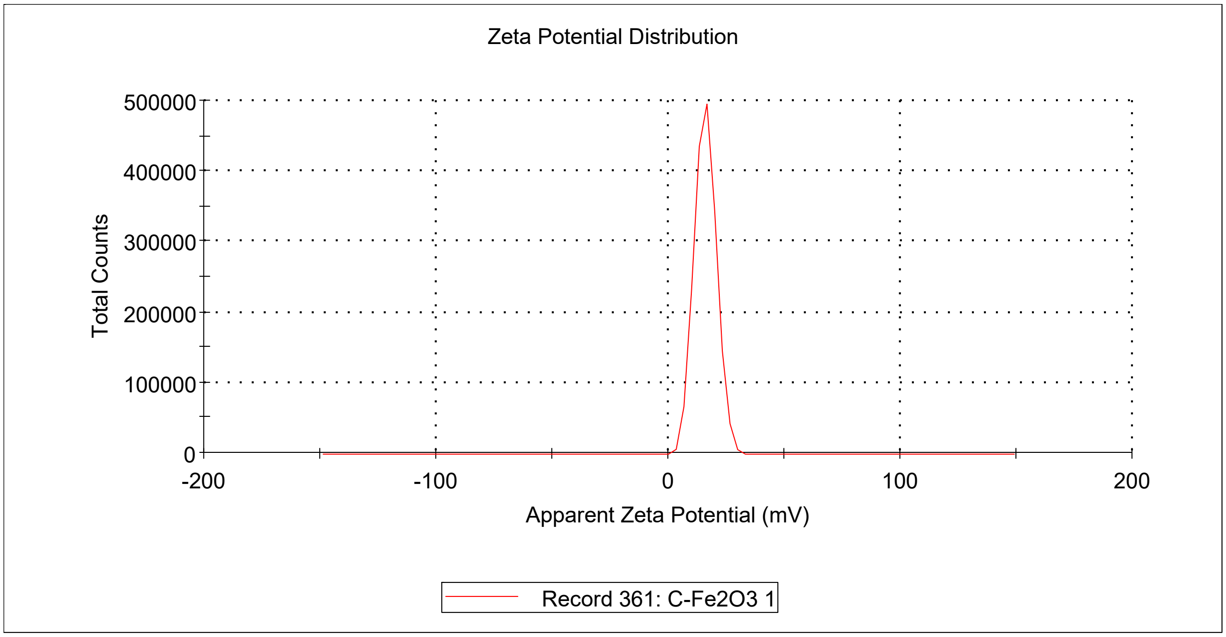

Supplement: Supplementary file 1 [file nanomaterials-15-01850-s001.zip › PDF S4.pdf]
